# Visualizing the intercity correlation of PM_2.5_ time series in the Beijing-Tianjin-Hebei region using ground-based air quality monitoring data

**DOI:** 10.1371/journal.pone.0192614

**Published:** 2018-02-13

**Authors:** Jianzheng Liu, Weifeng Li, Jiansheng Wu, Yonghong Liu

**Affiliations:** 1 Department of Urban Planning and Design, Faculty of Architecture, The University of Hong Kong, Hong Kong, China; 2 Shenzhen Institute of Research and Innovation, The University of Hong Kong, Shenzhen, China; 3 Key Laboratory of Human Environmental Science and Technology, Peking University Shenzhen Graduate School, Shenzhen, China; 4 Key Laboratory for Earth Surface Processes, College of Urban and Environmental Sciences, Peking University, Beijing, China; 5 School of Engineering, Sun Yat-Sen University, Guangzhou, China; Beihang University, CHINA

## Abstract

The Beijing-Tianjin-Hebei area faces a severe fine particulate matter (PM_2.5_) problem. To date, considerable progress has been made toward understanding the PM_2.5_ problem, including spatial-temporal characterization, driving factors, and health effects. However, little research has been done on the dynamic interactions and relationships between PM_2.5_ concentrations in different cities in this area. To address the research gap, this study discovered a phenomenon of time-lagged intercity correlations of PM_2.5_ time series and proposed a visualization framework based on this phenomenon to visualize the interaction in PM_2.5_ concentrations between cities. The visualizations produced using the framework show that there are significant time-lagged correlations between the PM_2.5_ time series in different cities in this area. The visualizations also show that the correlations are more significant in colder months and between cities that are closer, and that there are seasonal changes in the temporal order of the correlated PM_2.5_ time series. Further analysis suggests that the time-lagged intercity correlations of PM_2.5_ time series are most likely due to synoptic meteorological variations. We argue that the visualizations demonstrate the interactions of air pollution between cities in the Beijing-Tianjin-Hebei area and the significant effect of synoptic meteorological conditions on PM_2.5_ pollution. The visualization framework could help determine the pathway of regional transportation of air pollution and may also be useful in delineating the area of interaction of PM_2.5_ pollution for impact analysis.

## Introduction

The Beijing-Tianjin-Hebei area, which is the national capital region of China, is considered one of the most urbanized and developed areas in the country. However, despite its remarkable economic prosperity, it now has the reputation of being a “nuclear winter” region, as reported by the media, due to severe fine particulate matter (PM_2.5_) pollution [1]. Obviously, PM_2.5_ pollution not only undermines the reputation of the Beijing-Tianjin-Hebei area in terms of its economic prosperity, but more importantly, it also causes considerable public concern regarding health and poses critical challenges related to the sustainable development of cities within the region.

Substantial efforts have been made toward understanding PM_2.5_ pollution in China, including the spatial-temporal characterization [2–8], source apportionment[9, 10], influencing factors (e.g., meteorology) [7, 11–13], monitoring and mitigation policies [14–16] and health effects [17]. These efforts have greatly enriched the knowledge of PM_2.5_ pollution and have done a remarkable job in helping inform pollution mitigation policies. However, to date, little research has been done to investigate the dynamic relationships of PM_2.5_ concentrations in different cities at different times. In 2013, the Chinese State Council announced its action plan [18] to reduce the PM_2.5_ concentration in the Beijing-Tianjin-Hebei area by up to 25% by 2017, relative to the 2012 level. Some of the efforts include reducing emissions, building regional coordination mechanisms between local governments, etc. Such efforts, however, require investigations of the underlying associations of PM_2.5_ concentrations in different cities at different times in this area. A better understanding of the dynamic relationships and interactions of PM_2.5_ concentrations in different cities in the Beijing-Tianjin-Hebei area could help improve evidence-based practices in the action plan and help city managers in developing effective measures for pollution mitigation.

This study aims to address the aforementioned gap to allow a better understanding of the dynamic relationships between PM_2.5_ concentrations in different cities in the Beijing-Tianjin-Hebei area. Specifically, this paper presents visualizations of a significant phenomenon of time-lagged intercity correlations of PM_2.5_ time series based on ground-based air quality monitoring data. It is hoped that this study can contribute to the field in three aspects: (1) it proposes a visualization framework to visualize the intercity correlation of PM_2.5_ time series, and the visualization framework can be applied to other pollutants of interest; (2) it demonstrates the interactions of air pollution in cities of Beijing-Tianjin-Hebei area and the significant effect of synoptic meteorological conditions on air pollution, which provides evidence of visualization and corroborates previous studies on the interactions of air pollution and the effects of meteorological conditions on air pollution; and (3) the visualizations can help determine the pathway of regional transportation of air pollution and may also be useful in delineating the area of interaction of PM_2.5_ pollution for impact analysis.

The remainder of the article is organized as follows. The following section introduces the phenomenon of time-lagged intercity correlation of PM_2.5_ time series in different cities. The next section describes the visualization framework, the ground-based air quality monitoring data, and the wind vector data. The section of results and discussion presents the visualizations, their interpretations and implications, as well as their limitations. The final section concludes with a summary of the findings.

## What is the time-lagged intercity correlation of PM_2.5_ time series?

**Fig 1:**
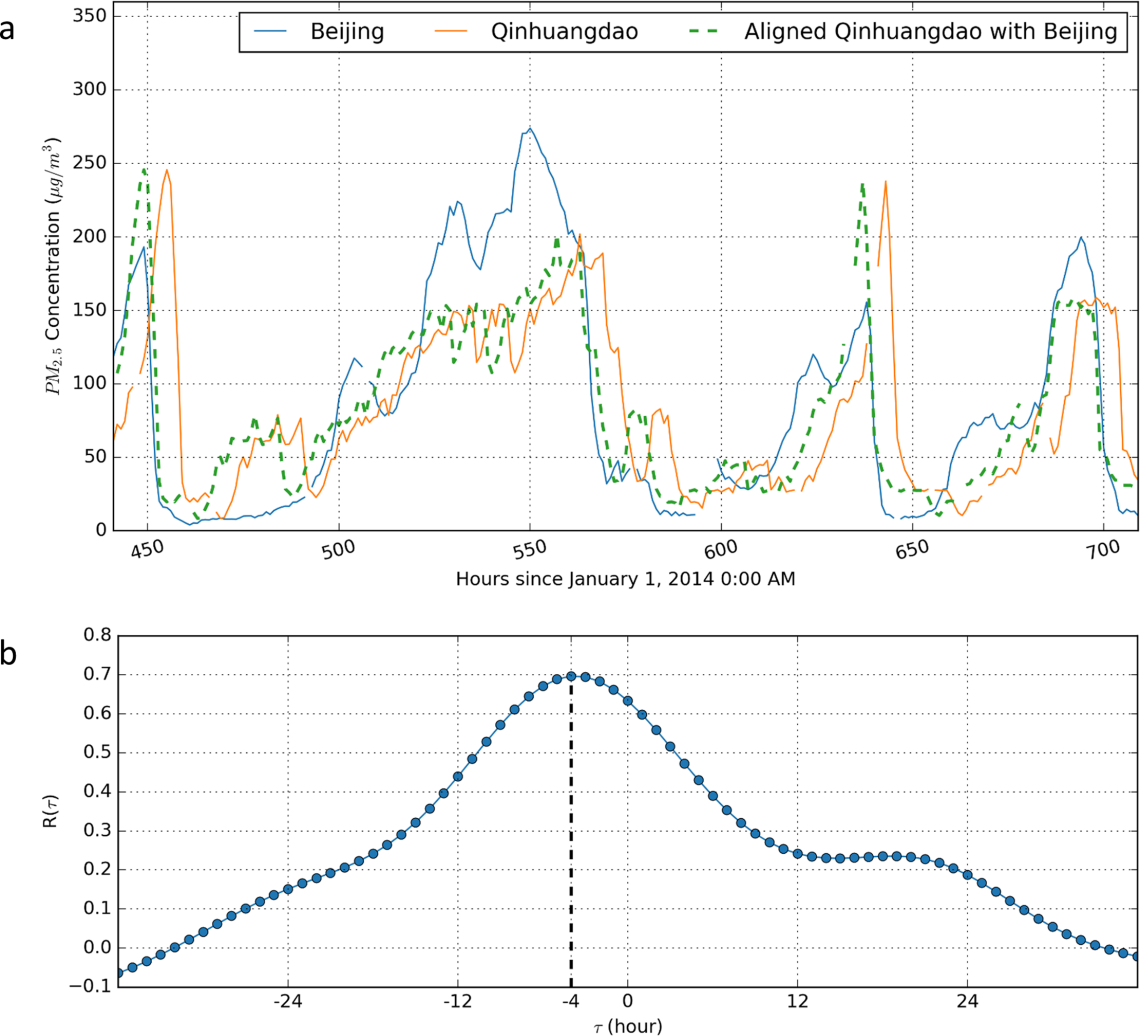
Cross-correlation of PM_2.5_ time series between Beijing and Qinhuangdao. **(A) An illustration of the time-lagged correlation of PM**_**2.5**_ **measurement time series between Beijing and Qinhuangdao.** The dashed line refers to the aligned PM_2.5_ time series in Qinhuangdao, which is shifted to the left by approximately 4 h to attain the best alignment with the PM_2.5_ time series in Beijing. Note that the gaps between the lines indicate missing data. **(B) Cross-correlation function R(τ).** Dots represent the calculated correlation coefficients based on varying time lags τ, while the solid line shows the variation of the correlation coefficients. Note that the cross-correlation function is at its maximum when the time lag τ is -4.

What this study attempts to visualize is a phenomenon that we defined as time-lagged intercity correlation of the PM_2.5_ time series. We have found that strong associations exist between PM_2.5_ time series in nearby cities when examining the patterns of the PM_2.5_ time series. The strength of the associations varies considerably between different cities and in different months. We also found that there are obvious time lags between PM_2.5_ time series in nearby cities. To illustrate this time-lagged intercity correlation relationship, an example is given using the Beijing and Qinhuangdao PM_2.5_ time series in January 2014. As seen in Fig 1A, the PM_2.5_ time series of Beijing and Qinhuangdao had very similar trends, and there was an obvious time delay between the two time series. It was easily found that their best alignment can be obtained by shifting the Qinhuangdao PM_2.5_ time series to the left by approximately 4 h.

As will be demonstrated later in the section on the results and discussion, the strong associations between PM_2.5_ time series of different cities not only exist in the case of Beijing and Qinhuangdao but also applies to many other cities. This phenomenon of intercity correlation of PM_2.5_ time series may be simple, but we believe that visualizing this phenomenon could generate useful and important insights regarding PM_2.5_ pollution which will be elaborated later.

## Data and methods

### Data

**Fig 2:**
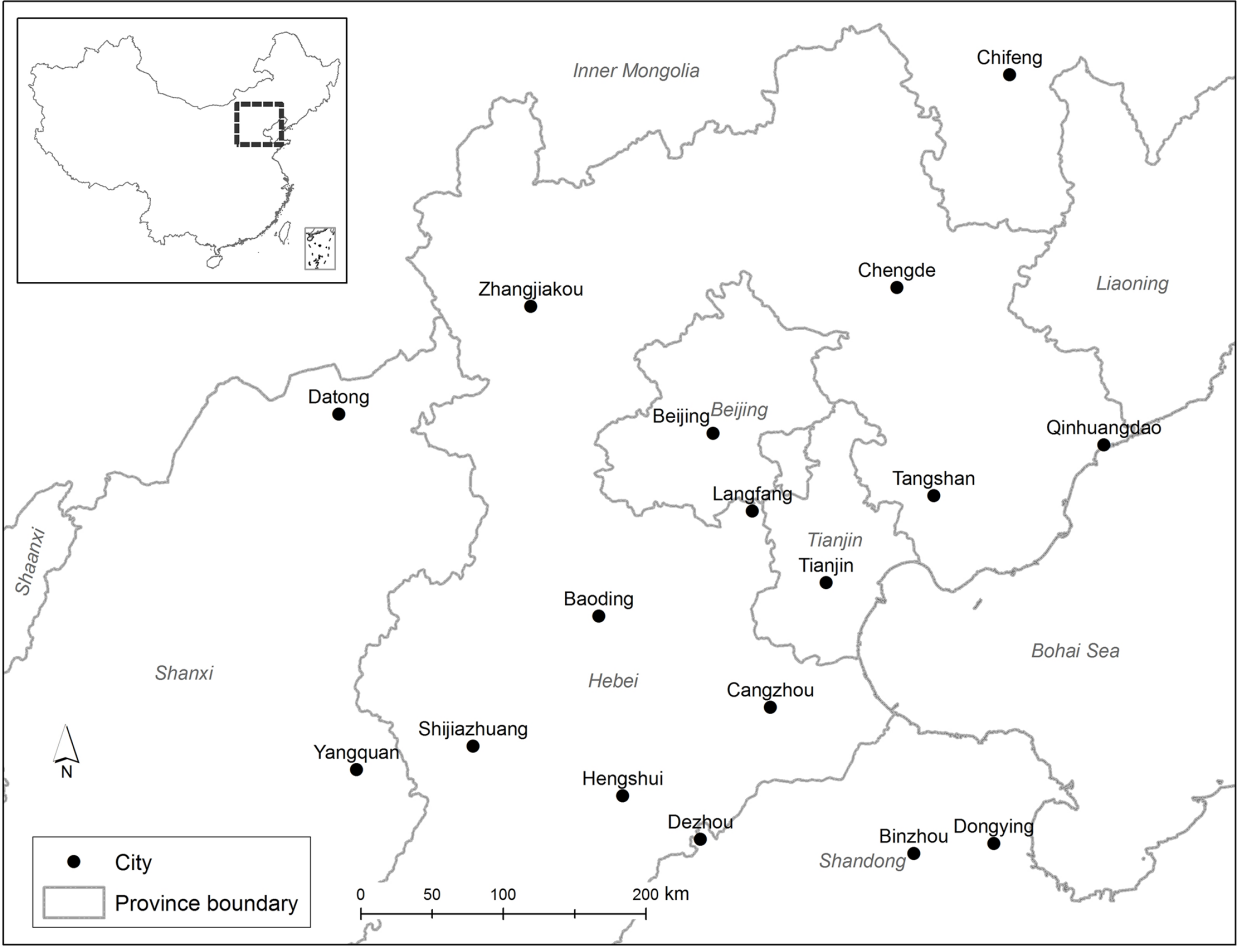
Cities in the Beijing–Tianjin–Hebei region. This map was generated using ArcGIS 10.2.2 (www.esri.com).

The Beijing-Tianjin-Hebei area is usually regarded as an economic region surrounding Beijing, Tianjin, and Hebei. This region encompasses nine cities, including Beijing, Tianjin, Baoding, Shijiazhuang, Tangshan, Cangzhou, Langfang, Zhangjiakou, and Chengde. However, in order to obtain an overview of the intercity correlation of PM_2.5_ series within this region, this study includes all 17 cities that are within approximately 360 km of Beijing. Therefore, this study also includes Qinghuangdao, Hengshui, Chifeng, Datong, Yangquan, Dongying, Binzhou, and Dezhou (Fig 2).

The PM_2.5_ measurement data from the Beijing-Tianjin-Hebei region used in this study were obtained from the national hourly air quality reporting platform (http://113.108.142.147:20035/emcpublish/) run by the China National Environment Protection Agency. These data consist of hourly concentrations of six major pollutants since early 2013: particulate matter with aerodynamic diameters no greater than 2.5 microns (PM_2.5_), particulate matter with aerodynamic diameters less than 10 microns (PM_10_), sulfur dioxide (SO_2_), nitrogen dioxide (NO_2_), ozone (O_3_), and carbon monoxide (CO). However, these data are not easily accessible, because the online reporting system only lists air quality information for the current day and historical data are unavailable to the public. Fortunately, third parties such as AQISTUDY.cn (https://www.aqistudy.cn/) and EPMAP.org (http://epmap.org) have been crawling these data from national hourly air quality reporting platform since late 2013.

This study used air quality monitoring data from 1 January 2014 to 31 December 2014 that were obtained by AQISTUDY.cn and EPMAP.org. There were missing hourly measurements in both data sources; therefore, we combined the two datasets to obtain a more complete 24-h PM_2.5_ measurement dataset for each day in 2014.

A comprehensive quality check of the raw data was conducted to reduce the impact of problematic data points, including duplicated data records, missing measurements with placeholders, and implausible zeros. In particular, data points with extremely high PM_2.5_ concentrations (>1000 μg/m^3^) were considered problematic outliers, and therefore, such data points were removed from the analysis.

Following the quality check, the air quality monitoring data were processed to facilitate the city-based cross-correlation analysis. First, for each city, the hourly PM_2.5_ concentration was calculated by averaging the hourly data from all stations within that city. Then, a linear interpolation was used to fill missing data points. Finally, a simple 8-h moving average was calculated to better capture the general trend of the PM_2.5_ time series and to reduce the impact of noise.

Wind vector data were used to help verify the effects of synoptic meteorological conditions on PM_2.5_ pollution. This study adopted the Modern-Era Retrospective Analysis for Research and Applications version 2 dataset (MERRA-2) produced by NASA. This dataset is an atmospheric reanalysis dataset from NASA that uses the Goddard Earth Observing System Model (version 5), which is based on atmospheric, land, and ocean observations from satellites, aircraft, and ships [19]. Specifically, this study used a monthly averaged atmospheric diagnosis product, i.e., M2TMNXSLV version 5.12.4 [20]. The key variables in this dataset consisted of eastward and northward wind speeds at 50 m above the surface, from which wind speed and wind direction were computed. These data are available from NASA Modern-Era Retrospective Analysis for Research and Applications version 2 dataset (MERRA-2) website (https://disc.gsfc.nasa.gov/datareleases/merra_2_data_release).

### Visualization framework

This study used a visualization framework to visualize the underlying dynamic interactions of PM_2.5_ time series in different cities based on the ground-based air quality monitoring data. The main component of the visualization framework is the cross-correlation method, which calculates intercity correlations between PM_2.5_ time series in different cities. In the following sections, this paper will introduce the cross-correlation method, the threshold guidance and significance test for the coefficients, and the implementation and presentation of the results.

#### Cross-correlation method

The cross-correlation method is a technique used in the field of signal processing to measure the similarity of two time series as a function of the lag of one relative to the other [21]. This technique is simple, but it has various applications including speech recognition, microphone-array processing [22], and even genetic studies [23]. This study used the cross-correlation method to determine the time delay between two PM_2.5_ time series. The calculation process consists of two principal steps: the calculation of the correlation coefficients between two time series at different time lags, and the selection of the time lag when the maximum correlation coefficient is reached. This maximum correlation coefficient occurs at the time shift for which the two time series are best aligned. The calculation process can be expressed using the following equations:

$$R(\tau )=Corr(S_{1}(t),S_{2}(t-\tau )),$$ (1)

$$T_{delay}={argmax}_{\tau }(R(\tau )),$$ (2)

where *S*_1_ and *S*_2_ are the two time series to be computed, *τ* is the time lag, *R*(*τ*) is the correlation coefficient calculated between *S*_1_ and *S*_2_ when the time lag is *τ*, *argmax*_*τ*_ refers to the argument (in this case, the time lag *τ*) at which the values of the function *R*(*τ*) are maximized. *T*_*delay*_ denotes the time lag that generates the maximum correlation coefficient *R*_*max*_.

To illustrate the cross-correlation analysis, this study used the Beijing and Qinghuangdao PM_2.5_ time series in January 2014 as an example. First, the correlation coefficients were calculated at different time lags; then, the maximum correlation coefficient was identified; and finally, the time lag that created the maximum correlation coefficient was determined. Fig 1B shows the maximum correlation coefficient occurs when the PM_2.5_ time series from Qinhuangdao is shifted to the left by approximately 4 h. Therefore, the time lag that attained the best alignment and created the maximum correlation coefficient was determined as 4 h (Fig 1B). As seen from Fig 1A, the best alignment between the two time series was obtained by shifting the Qinhuangdao PM_2.5_ time series to the left by approximately 4 h, which is consistent with the calculation from the cross-correlation method.

#### Threshold guidance and significance testing of coefficients

The maximum correlation coefficient in the example above is 0.697 (Fig 2B), which suggests a reasonably strong correlation between the PM_2.5_ time series in Beijing and Qinhuangdao. However, not all of the maximum correlation coefficients for PM_2.5_ time series between each pair of cities attained such a desirable correlation. In some cases, the correlation of the PM_2.5_ time series was very low, which indicated that the PM_2.5_ time series were not correlated at all. Therefore, correlation coefficient thresholds were needed to distinguish relationships that were correlated from those that were not. This study used a rule of thumb proposed by Dennis Hinkle and his coauthors [24] to interpret correlation coefficients. Specifically, correlation coefficients of 0.7–0.9 and 0.9–1.0 were considered high and very high correlations, respectively. A coefficient of 0.5–0.7 was considered a moderate correlation. Coefficients of 0.3–0.5 and 0.0–0.3 were regarded as having a low or little correlation, respectively. In this study, correlation coefficients >0.5 were considered indicative of a probable correlated relationship and coefficients <0.5 were regarded as uncorrelated relationships.

As shown above, time lags for each month were determined by computing the maximum correlation coefficient, *R*_*max*_, based on the ground-based air quality monitoring data in 2014. Tests of significance were needed to examine whether the maximum correlation coefficient, *R*_*max*_, was significantly larger than the correlation coefficient without the time lag. The correlation coefficient without the time lag is denoted as *R*(0) here. A value of *R*_*max*_ that was significantly larger than *R*(0) indicates that the difference between the two coefficients is not due to random chance and that it is safe to use the time lag, *T*_*delay*_, and the maximum correlation coefficients for further analysis. To test the difference between two correlations, the correlations were transformed using Fisher’s r-to-z transformation [25]. Details about this transformation and calculation are illustrated in [26].

#### Implementation and presentation

The cross-correlation method and the significance tests for coefficients are implemented using Python 2.7.5 (https://www.python.org). All figures were drawn using Python 2.7.5 and Matplotlib 1.5.0 (https://matplotlib.org/).

To provide a clear and intuitive presentation on the interactions between PM_2.5_ time series in different cities, this study employed two different forms to display the results. One is a map presentation, and the other is a matrix presentation. The map presentation offers a geographic representation of the dynamic relationships between PM_2.5_ time series in different cities, while the matrix presentation provides a tabulation of these associations.

## Results and discussion

### Interpretation and explanation

**Fig 3:**
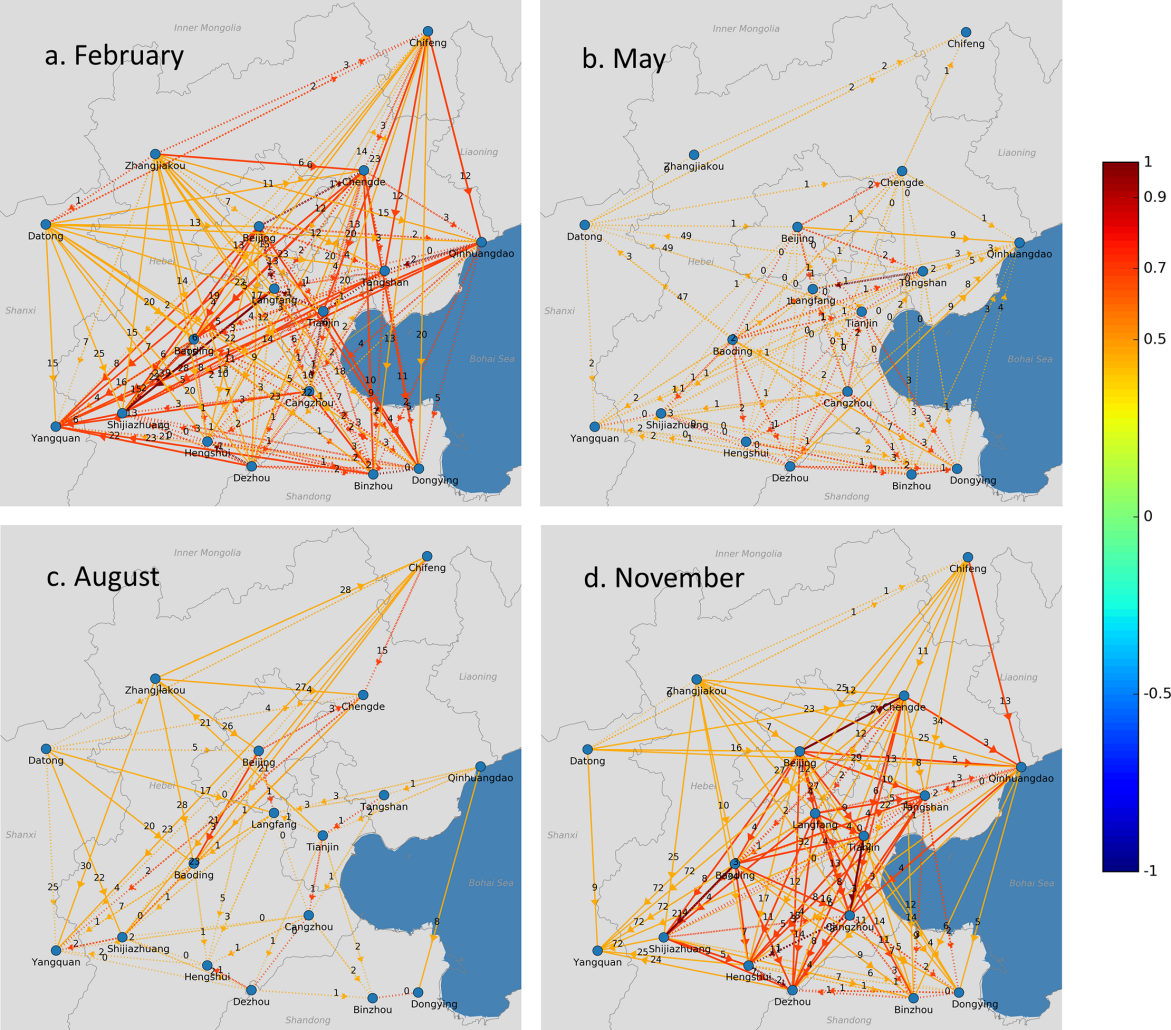
**Maps of intercity correlations and time lags for PM**_**2.5**_ **time series between 17 cities in (A) February, (B) May, (C) August, and (D) November of 2014 in the Beijing-Tianjin-Hebei area.** The line colors refer to the strength of the correlation of the PM_2.5_ time series between the two linked cities. The color bar on the right provides a scale of the correlation coefficients. The numbers on the lines refer to the time lags. Solid lines mean the correlation with the time lag is significantly larger than the correlation without the time lag at the 10% level, while the dashed lines mean the correlation with the time lag is not significantly larger than the correlation without the time lag at the 10% level. The arrows on the lines indicate the temporal order of the correlated PM_2.5_ time series, where the PM_2.5_ time series in the city at the tail of the arrow leads the PM_2.5_ time series in the city at the head of the arrow. Map views for other months are provided in the supplementary information (S1 Fig). This figure was produced using Python 2.7.5 (https://www.python.org) and Matplotlib 1.5.0 (https://matplotlib.org/).

**Fig 4:**
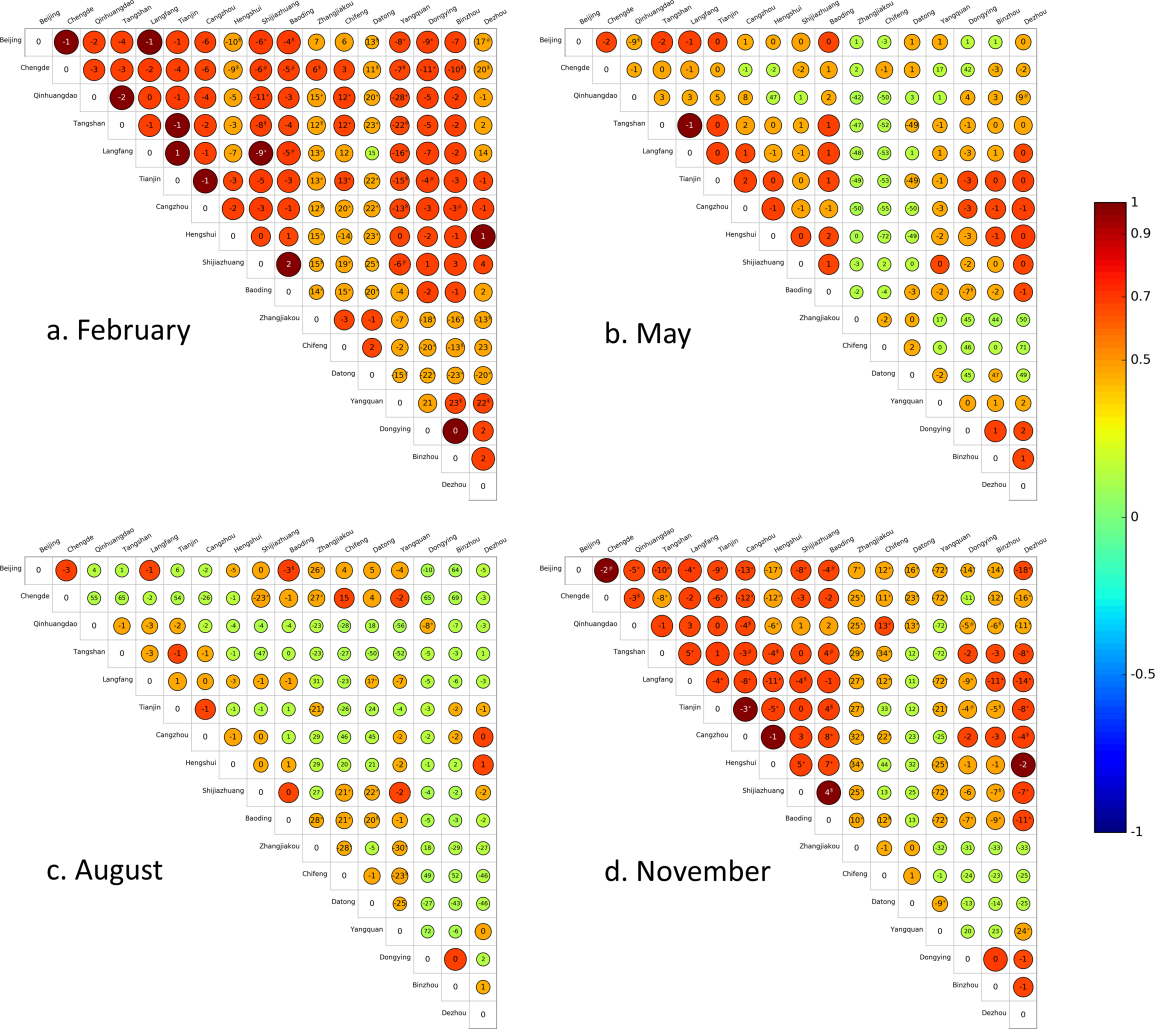
**Matrix views of the intercity correlations and time lags for PM**_**2.5**_ **time series between 17 cities in (A) February, (B) May, (C) August, and (D) November of 2014 in the Beijing-Tianjin-Hebei area.** The color and size of the circles indicate the strength of the correlation. The color bar on the right provides a scale of the correlation coefficients. Orange, red, and claret colors mean the correlation coefficients are over 0.5, 0.7, and 0.9, respectively, while the green color indicates correlation coefficients <0.5. The label within the circle refers to the time lag. A positive time lag, τ, means the city on the y-axis lags the city on the x-axis by τ hours. Similarly, a negative time lag, τ, means the city on the y-axis leads the city on the x-axis by -τ hours. A hash sign (#) means the correlation for the time lag is significantly larger than the correlation without the time lag at the 10% level. A dollar sign ($) refers to the statistical significance at the 5% level. An asterisk (*) refers to the statistical significance at the 1% level. Note that for clear presentation, all matrix views show only the upper portion of the matrix to avoid duplication. Matrix views for other months are provided in the supplementary information (S2 Fig). This figure was produced using Python 2.7.5 (https://www.python.org) and Matplotlib 1.5.0 (https://matplotlib.org/).

The cross-correlation analysis was performed on a monthly basis using the ground-based PM_2.5_ air quality monitoring data in 2014. For clarification, we visualized the results for February, May, August, and November using a map view (Fig 3) and a matrix view (Fig 4). The visualizations of results for all 12 months in 2014 are presented in the supplementary information (S1 Fig and S2 Fig). In Fig 3, the line colors indicate the strength of the correlation between the two PM_2.5_ time series. The labels in the lines denote the time lags, and the arrows show the temporal order of the correlated PM_2.5_ time series. The circle colors in Fig 4 match the color scheme in Fig 3.

As seen in Figs 3 and 4, and S1 and S2 Figs, visualizations using the empirical ground-based air quality monitoring data show that significant correlations exist between PM_2.5_ time series from different cities in the Beijing-Tianjin-Hebei area, and that the correlations between different cities attain peaks at different time lags. Moreover, these intercity correlations and their associated time lags vary considerably depending on season and location. Usually, the correlations were more significant in colder months (e.g., February and November) and between cities that are closer. Furthermore, there were seasonal changes of the signs of the time lags, indicating seasonal changes in the temporal order for correlated PM_2.5_ time series. As seen in Fig 3 and S1 Fig, most of the arrows point toward the south in colder months, such as February and November, while in warmer months there are plenty of arrows pointing toward the north. This means that in colder months, the PM_2.5_ time series in cities on the north side of the region lead the PM_2.5_ time series in cities on the south side, while in warmer months, the PM_2.5_ time series in cities on south side may lead those in cities on the north side. These insights suggest that air pollutants in the Beijing-Tianjin-Hebei area are interacted with each other, which is consistent with and supported by previous studies [3, 4] that have found the existence of strong bidirectional coupling between Beijing and neighboring cities.

**Fig 5:**
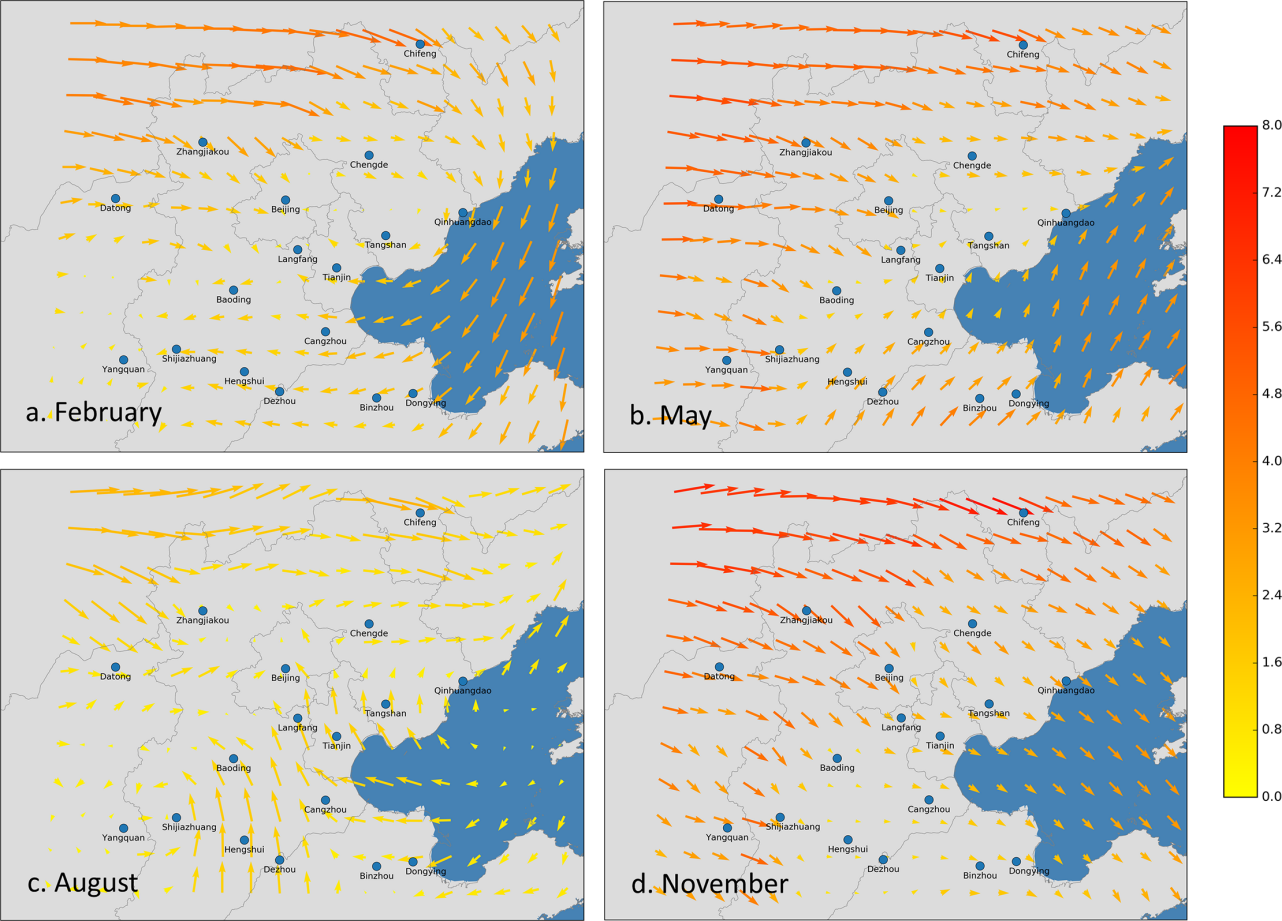
**Time-averaged wind vectors 50 m above the surface in (A) February, (B) May, (C) August, and (D) November of 2014 in the Beijing-Tianjin-Hebei area.** The length and color of the arrows are both indicative of wind speed. The color bar on the right provides a scale of the wind speed (unit: m/s). This figure is produced using Python 2.7.5 (https://www.python.org) and Matplotlib 1.5.0 (https://matplotlib.org/).

The aforementioned seasonal patterns and associations also suggest that the intercity correlations of PM_2.5_ time series are closely related to synoptic meteorological conditions. In fact, we believe that these intercity correlations are almost certainly due to synoptic meteorological variations. These meteorological conditions, such as wind and air pressure, control the passage of air masses (e.g., cold fronts). The passage of air masses further influences the variation in PM_2.5_ concentrations [27, 28], which makes the variation in PM_2.5_ concentrations in cities along the path of the passage similar to each other. In colder months, meteorological conditions including wind speeds and cold fronts are stronger than those during the warmer months; thus, the effects of meteorological conditions on PM_2.5_ concentrations are stronger during colder months, making the intercity correlations of PM_2.5_ time series much more significant during colder months. In addition, we found that the temporal order of the correlated PM_2.5_ time series match the wind vectors very well. As shown in Fig 5, the directions of the time lags in Fig 3 are generally consistent with the wind vectors. The wind also explains the seasonal changes in the temporal order of the correlated PM_2.5_ time series because of the seasonal wind change in north China. This finding corroborated previous studies [2, 7, 8, 11, 12, 28–30] showing that meteorological conditions play a significant role in affecting PM_2.5_ concentrations.

### Implications

The visualizations in this paper show the significant interactions of PM_2.5_ concentrations between nearby cities. This means policies that did not take the interactions of PM_2.5_ concentrations into account might be problematic. The corridor building policy in Beijing which proposed to build five 500 m wide ventilation corridors to “blow the pollution away from Beijing and let it harm other cities” [31], for example, is probably not appropriate. In addition, the significant interaction between PM_2.5_ concentrations also suggests that it is unnecessary for government authorities and urban residents to accuse each other [32, 33], as air pollution in nearby cities significantly interacts with each other.

The visualization framework presented in this study could be used in several potential applications. First, as seen in Figs 3 and 5, the temporal order and the time lags of the correlated PM_2.5_ time series that were identified by the framework could be used to verify paths of regional air pollution transportation. Although there are other approaches that identify pathways, such as backward trajectory modeling [10, 34] or a chemical transport modeling system [35, 36], the time-lagged intercity correlations found in this study, which are due to synoptic meteorological processes, may serve as additional evidence for determining the potential pathways of regional air pollution transportation.

Second, significant correlations of the PM_2.5_ time series between cities and their strengths provided information which might be useful in delineating the area of interaction of the PM_2.5_ pollution, where the PM_2.5_ concentrations interact with each other. The delineation of the area of interaction could be further used to help define management zones for air pollution control and in impact analysis to assess environmental, economic and health losses. Third, the visualization framework presented in this study can be applied to data for other pollutants as well as the optical and chemical properties of air pollutants to explore their underlying dynamic links between cities.

### Limitations

This research proposes a visualization framework to visualize the intercity correlations of PM_2.5_ pollution and provides an overview of the interactions between air pollution in different cities in the Beijing-Tianjin-Hebei region. The results may help with the identification of regional air pollution transport pathways, as well as the delineation of the area of interaction of PM_2.5_ pollution. Despite these contributions, this study has its limitations.

First, the visualizations in this study are able to reveal patterns in PM_2.5_ pollution as visualization studies usually do, but they cannot answer questions regarding the cause and inner mechanisms of pollution in a complicated atmospheric environment (e.g., how intercity associations develop with climate change and emissions mitigation, and how various synoptic meteorological conditions affect these associations. More research is needed to address these questions.

Second, our study only used twelve month data to explore the patterns in intercity correlations of PM_2.5_ time series. The seasonal patterns on the temporal order for correlated PM_2.5_ time series might be subject to annual variation in PM_2.5_ pollution. However, we speculate that due to the dominant influence of synoptic meteorological conditions on PM_2.5_ pollution, this seasonal pattern would persist. However, for cautionary purposes, we advise that future research is needed to confirm this pattern.

## Conclusion

This study discovered a phenomenon of time-lagged intercity correlations of PM_2.5_ time series and proposed a visualization framework based on this phenomenon to visualize the interactions in PM_2.5_ concentrations between cities. Using this framework, this study visualized the intercity correlation of PM_2.5_ time series between cities in the Beijing-Tianjin-Hebei region. The visualization results show that significant correlations exist between PM_2.5_ time series of different cities in this region, and correlations are more significant in colder months and between cities that are closer. The visualizations also show that there are seasonal changes in the temporal order of the correlated PM_2.5_ time series. Further analysis suggests that the intercity correlations of PM_2.5_ time series are probably due to synoptic meteorological variations, which corroborate with previous studies. In addition to the visualization framework, which can be used in several potential applications, the major contribution of this study is that the visualizations revealed the significant underlying dynamic relationships of PM_2.5_ concentrations between cities and provided visual evidence for interactions of air pollution between nearby cities.

## Supporting information

S1 Fig Maps of intercity correlations and time lags for the PM_2.5_ time series between 17 cities within the Beijing-Tianjin-Hebei area in 2014. (JPG)

S2 Fig Matrix views of the intercity correlations and time lags of the PM_2.5_ time series between 17 cities within the Beijing-Tianjin-Hebei area in 2014. (JPG)
