# Supplementary figures and images for: Visualizing the intercity correlation of PM2.5 time series in the Beijing-Tianjin-Hebei region using ground-based air quality monitoring data
Source: PLoS One. 2018 Feb 13;13(2):e0192614. doi: 10.1371/journal.pone.0192614 (PMC5811218; doi:10.1371/journal.pone.0192614)

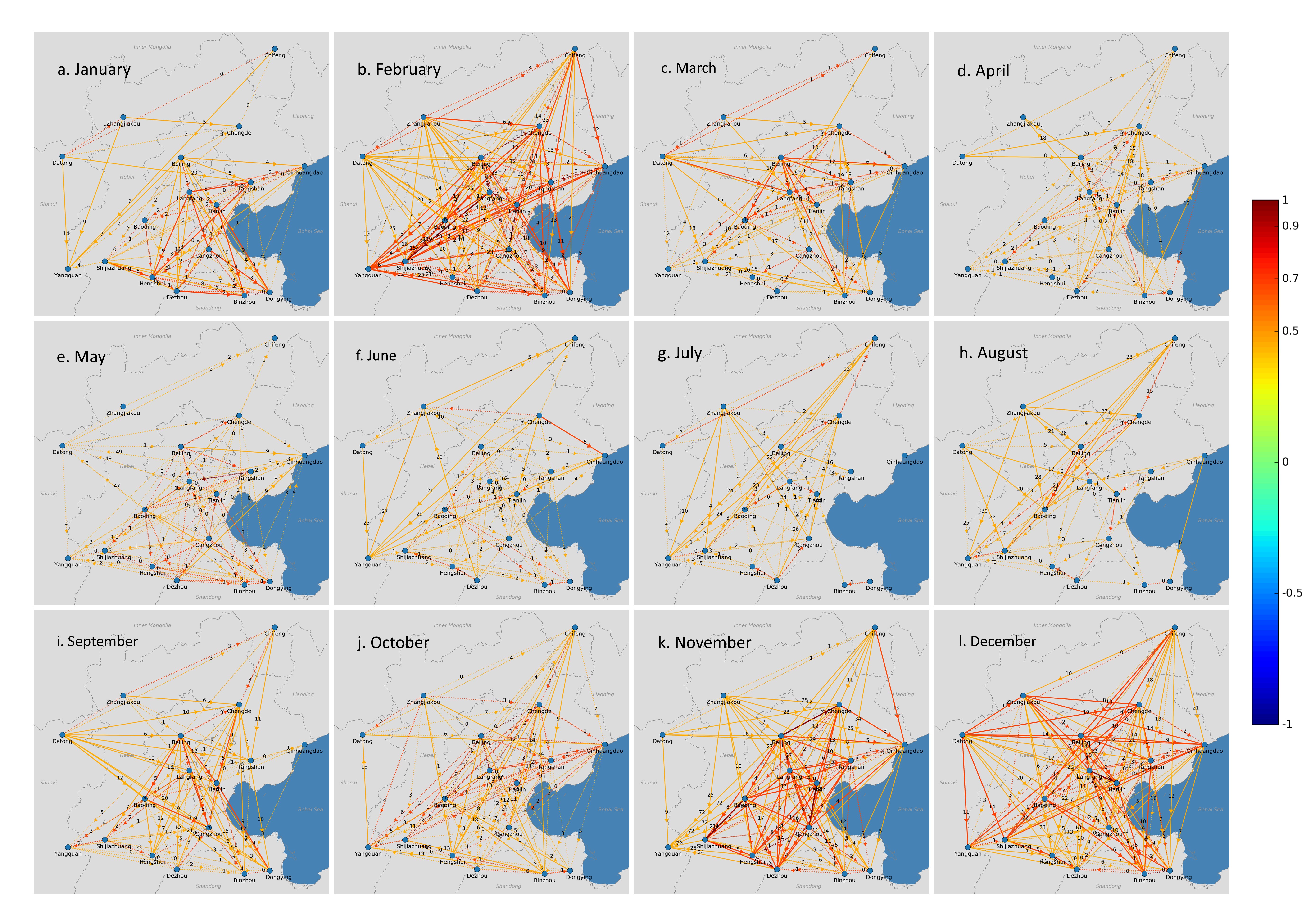

Supplement: S1 Fig — (JPG) [file pone.0192614.s001.jpg]

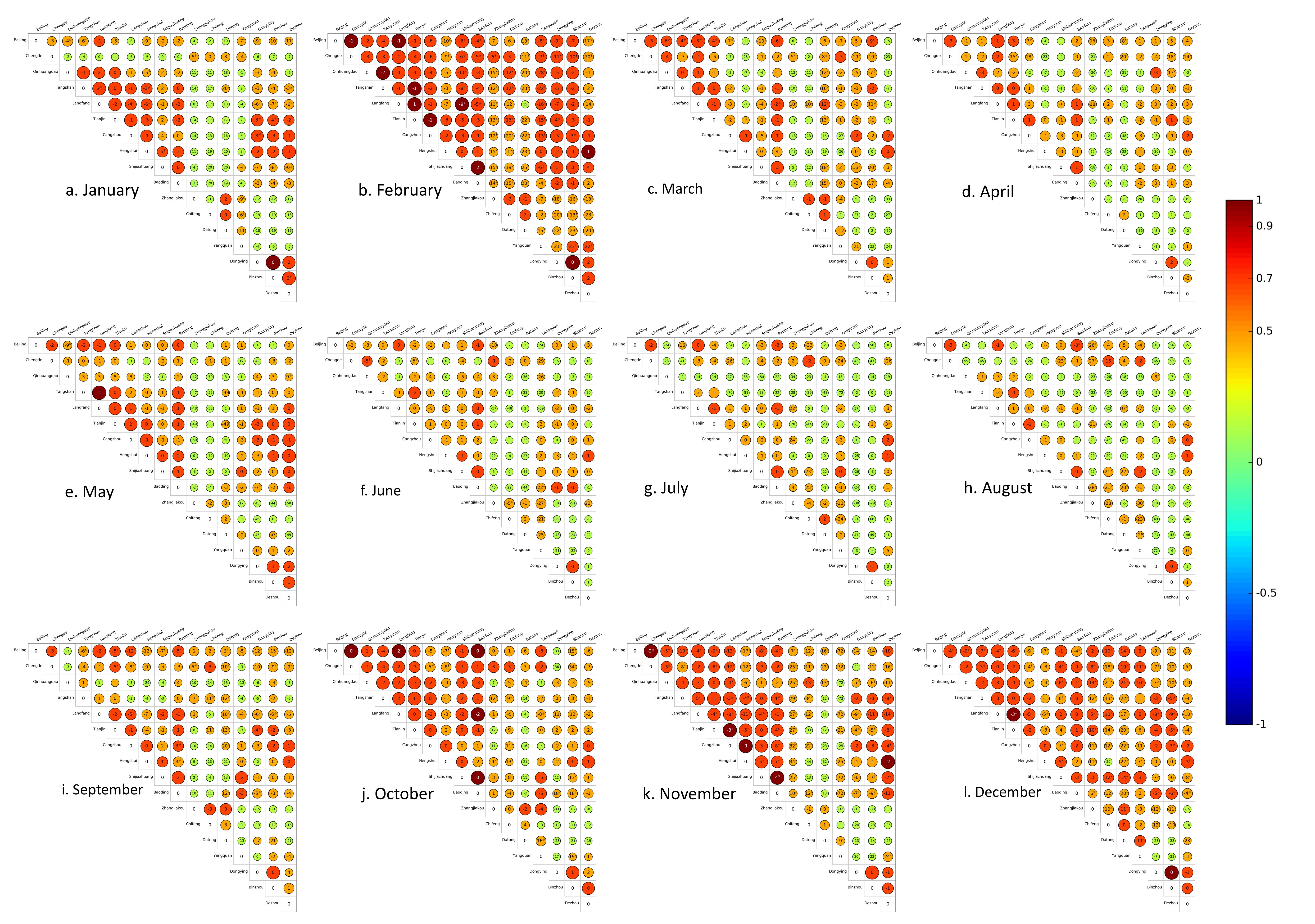

Supplement: S2 Fig — (JPG) [file pone.0192614.s002.jpg]
